# 依托泊苷联合G-CSF与环磷酰胺联合G-CSF在多发性骨髓瘤患者中进行自体外周血干细胞动员的比较

**DOI:** 10.3760/cma.j.cn121090-20231124-00274

**Published:** 2024-04

**Authors:** 国蓉 王, 光忠 杨, 传营 耿, 芸 冷, 垠 吴, 爱军 刘, 文明 陈

**Affiliations:** 首都医科大学附属北京朝阳医院血液科，北京市多发性骨髓瘤医疗研究中心，北京 100020 Department of Hematology, Beijing Chaoyang Hospital Affiliated to Capital Medical University, Beijing Medical Research Center for Multiple Myeloma, Beijing 100020, China

**Keywords:** 依托泊苷, 多发性骨髓瘤, 自体造血干细胞动员, 自体造血干细胞移植, Etoposide, Multiple myeloma, Autologous hematopoietic stem cell mobilization, Autologous hematopoietic stem cell transplantation

## Abstract

**目的:**

比较依托泊苷（ETO）联合G-CSF与环磷酰胺（CTX）联合G-CSF在多发性骨髓瘤（MM）患者中进行自体外周血造血干细胞动员的效果及安全性。

**方法:**

纳入2020年1月1日至2023年7月31在首都医科大学附属北京朝阳医院血液科接受自体外周血造血干细胞动员、采集的MM患者，利用倾向性评分按照1∶1匹配比例筛选出134例患者，ETO联合G-CSF动员方案（ETO组）、CTX联合G-CSF动员方案（CTX组）各67例，对其临床资料进行回顾性分析。

**结果:**

①ETO组、CTX组采集天数分别为2（1～3）d、2（1～5）d（*P*<0.001），CD34^+^细胞采集量分别为7.62（2.26～37.20）×10^6^/kg、2.73（0.53～9.85）×10^6^/kg（*P*<0.001），采集成功率分别为100.0％（67/67）、76.1％（51/67）（*P*<0.001）、采集优良率分别为82.1％（55/67）、20.9％（14/67）（*P*<0.001）。ETO组有2例患者在采集1 d后进行方案转换，CTX组有11例患者在采集1～2 d后进行方案转换。②ETO组、CTX组粒缺伴发热发生率分别为21.5％（14/65）、10.7％（6/56）（*P*＝0.110），血小板输注患者占比分别为10.7％（7/65）、1.8％（1/56）（*P*＝0.047）。③至随访截止，ETO组63例、CTX组54例患者接受了自体造血干细胞移植，中位CD34^+^细胞回输量分别为4.62（2.14～19.89）×10^6^/kg、2.62（1.12～5.31）×10^6^/kg（*P*<0.001），中性粒细胞植入时间分别为11（9～14）d、11（10～14）d（*P*＝0.049），血小板植入时间分别为11（0～19）d、12（0～34）d（*P*＝0.035）。CTX组有1例患者发生血小板延迟植入。

**结论:**

依托泊苷联合G-CSF的动员方案可能有较多的患者需要输注血小板，但采集天数缩短，采集成功率、优良率及CD34^+^细胞采集量较高，移植后中性粒细胞和血小板植入较快。

## Abstract (en)

**Objective:**

The effect and safety of etoposide combined with G-CSF were compared with those of cyclophosphamide combined with G-CSF in autologous peripheral blood mobilization in patients with multiple myeloma（MM）.

**Methods:**

Patients with MM who received autologous peripheral blood stem cell mobilization and collection in the Department of Hematology, Beijing Chaoyang Hospital Affiliated to Capital Medical University from January 1, 2020 to July 31, 2023 were included. A total of 134 patients were screened by propensity score matching technology according to a 1∶1 ratio. A total of 67 cases were each treated with ETO combined with G-CSF mobilization scheme（ETO group）and CTX combined with G-CSF mobilization scheme（CTX group）. Their clinical data were retrospectively analyzed.

**Results:**

①Collection results: the ETO and CTX groups［2（1–3）d *vs* 2（1–5）d; *P*<0.001］and CD34^+^ cells［7.62×10^6^（2.26×10^6^–37.20×10^6^）/kg *vs* 2.73×10^6^（0.53×10^6^–9.85×10^6^）/kg; *P*<0.001］were collected. The success rate of collection was 100.0％（67/67）versus 76.1％（51/67）（*P*<0.001）. Excellent rate of collection was 82.1％（55/67）*vs* 20.9％（14/67; *P*<0.001）. Two patients in the ETO group switched protocols after 1 day of collection, and 11 patients in the CTX group switched protocols after 1–2 days of collection. ②Adverse reactions: granular deficiency with fever［21.5％（14/65）*vs* 10.7％（6/56）; *P*＝0.110］, requiring platelet transfusion［10.7％（7/65）*vs* 1.8％（1/56）; *P*＝0.047］. ③Until the end of follow-up, 63 cases in the ETO group and 54 cases in the CTX group have undergone autologous transplantation. The median number of CD34^+^ cells infused in the two groups was 4.62×10^6^（2.14×10^6^–19.89×10^6^）/kg versus 2.62×10^6^（1.12×10^6^–5.31×10^6^）/kg（*P*<0.001）, neutrophil implantation time was 11（9–14）d versus 11（10–14）d（*P*＝0.049）, and platelet implantation time was 11（0–19）d *vs* 12（0–34）d（*P*＝0.035）. One case in the CTX group experienced delayed platelet implantation.

**Conclusion:**

The mobilization scheme of etoposide combined with G-CSF requires relatively platelet transfusion, but the collection days are shortened. The collection success rate, excellent rate, and the number of CD34^+^ cells obtained are high, and the neutrophil and platelet engraftment is accelerated after transplantation.

多发性骨髓瘤（MM）是最常见的血液系统恶性肿瘤之一，而且随着人口老龄化，发病率有逐渐上升的趋势[1],[2]。新药时代，蛋白酶体抑制剂、免疫调节剂、单抗类靶向药物、免疫治疗等，显著延长了MM的无病生存（DFS）甚至总生存（OS）期，明显改善了MM的预后[3]。但迄今为止，自体造血干细胞移植仍然是MM整体治疗中非常重要的环节，其地位尚不能被其他治疗取代[4]。而进行自体造血干细胞移植的前提条件之一就是通过造血干细胞动员、采集获得足够的自体造血干细胞。化疗联合G-CSF动员（简称化疗动员）是常用的动员方法，但目前MM尚无公认的特异性化疗动员方案。另一方面，普乐沙福虽然对化疗动员的地位构成一定影响，但其昂贵的价格限制了其临床应用。因此，有必要探索经济、安全、高效化疗的动员方案。我中心2010至2020年期间主要采用环磷酰胺（CTX）进行化疗动员，采集成功率、优良率分别为75.7％～91.7％、25.7％～55.6％[5]。为进一步提升造血干细胞动员的效果，我中心自2020年开始探索依托泊苷（ETO）化疗动员。本研究对2020年1月至2023年7月期间在本中心进行自体外周血干细胞动员、采集的MM患者进行配对分析，比较ETO和CTX两种化疗动员方案的采集效果。

## 病例与方法

一、病例

本项回顾性研究纳入2020年1月至2023年7月期间在首都医科大学附属北京朝阳医院采用ETO联合G-CSF（ETO组）和CTX联合G-CSF（CTX组）进行自体外周血干细胞动员、采集的MM患者，利用倾向性评分（PSM）按照1∶1比例匹配以减少组间的偏差，匹配因素包括性别、年龄、DS分期、含来那度胺化疗、含达雷妥尤单抗化疗、动员前疗程、动员前>6疗程治疗，共纳入134例MM患者，ETO组、CXT组各67例。两组患者的其他主要基线临床特征见表1。所有患者的诊断、分型、分期及疗效判断均符合《中国多发性骨髓瘤诊治指南（2022年修订）》[6]。本研究获得首都医科大学附属北京朝阳医院伦理委员会的批准（批件号：2022-科-493）。

**表1:** 依托泊苷（ETO）组与环磷酰胺（CTX）组多发性骨髓瘤患者临床特征比较

指标	ETO组（67例）	CTX组（67例）	统计量	*P*值
性别［例（%）］			*χ*^2^＝2.011	0.156
男	45（67.2）	37（55.2）		
女	22（32.8）	30（44.8）		
年龄［岁，*M*（范围）］	56（39~66）	55（29~64）	*z*＝−1.072	0.284
类型［例（%）］			*χ*^2^＝3.636	0.603
IgG	32（47.8）	28（41.8）		
IgA	20（29.8）	17（25.3）		
IgD	3（4.5）	2（3.0）		
轻链型	11（16.4）	16（23.9）		
不分泌型	1（1.5）	3（4.5）		
双表型	0	1（1.5）		
DS分期［例（%）］			*χ*^2^＝1.800	0.407
Ⅰ期	4（6.0）	2（3.0）		
ⅡA/ⅡB期	12（17.9）	8（11.9）		
ⅢA/ⅢB期	51（76.1）	57（85.1）		
R-ISS分期［例（%）］			*χ*^2^＝0.052	0.974
Ⅰ期	12（17.9）	13（19.4）		
Ⅱ期	41（61.2）	40（59.7）		
Ⅲ期	14（20.9）	14（20.9）		
含来那度胺化疗［例（%）］	59（88.1）	53（79.1）	*χ*^2^＝1.958	0.162
含达雷妥尤单抗化疗［例（%）］	7（10.4）	6（9.0）	*χ*^2^＝0.085	0.770
动员前化疗疗程［个，*M*（范围）］	4（3~17）	4（2~21）	*z*=−1.102	0.270
动员前化疗>6疗程［例（%）］	3（4.5）	4（6.0）	*χ*^2^＝0.151	0.698
动员前疾病状态评估［例（%）］			*χ*^2^＝3.408	0.333
CR	16（23.9）	18（26.9）		
VGPR	31（46.2）	21（31.3）		
PR	16（23.9）	23（34.3）		
<PR	4（6.0）	5（7.5）		

**注** CR：完全缓解；VGPR：非常好的部分缓解；PR：部分缓解

二、诱导治疗方案

参照国内外MM诊治指南[6]–[7]，初诊患者采用含硼替佐米/来那度胺等新药的诱导治疗方案，高危患者联合达雷妥尤单抗进行诱导治疗，伴髓外瘤患者采用含阿霉素或脂质体阿霉素方案诱导化疗。复发患者根据既往治疗、身体状况选择治疗方案。初诊患者诱导治疗4个疗程、复发患者在再诱导治疗获得疾病稳定或部分缓解（PR）及以上疗效时行自体外周血干细胞动员、采集。

三、自体外周血干细胞动员方案

ETO 375 mg·m^−2^·d^−1^×2 d或CTX 1.5 g·m^−2^·d^−1^×2 d，化疗结束第5天开始G-CSF 10 µg/kg每日1次皮下注射，至采集结束停药。

采集1～2 d未获得采集成功的患者更换其他方案或加用普乐沙福（转换方案）。普乐沙福用法为20 mg（患者体重≤83 kg）或24 mg（患者体重>83 kg），采集前晚22时皮下注射。

采集物行单个核细胞（MNC）计数，用流式细胞术检测CD34^+^细胞百分比及计数。CD34^+^细胞采集量≥2.0×10^6^/kg定义为采集成功，≥5.0×10^6^/kg定义为采集优良。

四、自体造血干细胞移植预处理方案及植入标准

采用美法仑200 mg/m^2^进行预处理，自WBC<2.5×10^9^/L起予以G-CSF 300 µg/d皮下注射，至中性粒细胞植入停药。植入标准：中性粒细胞计数（ANC）≥0.5×10^9^/L连续3 d为中性粒细胞植入；PLT≥20×10^9^/L连续7 d且脱离血小板输注为血小板植入。

五、不良反应评估

采用CTCAE5.0版分级标准对干细胞动员、采集及自体造血干细胞移植期间的不良反应进行评估。

六、统计学处理

采用SPSS 22.0软件进行描述性统计分析。组间临床差异和移植后造血重建时间采用卡方检验、*t*检验或非参数检验进行比较，采集量的影响因素采用偏相关分析或多因素回归分析。以*P*<0.05为差异有统计学意义。

## 结果

一、诱导化疗方案

ETO组诱导化疗方案：61例（91.0％）采用三药方案，其中54例（80.6％）采用含蛋白酶体抑制剂+免疫调节剂方案［VRD方案（硼替佐米+来那度胺+地塞米松）50例，74.6％］；6例（9.0％）采用含1种新药方案。6例（9.0％）采用四药方案（≥2种新药），以达雷妥尤单抗+VRD方案（D-VRD方案）应用最多（3例，4.5％）。

CTX组患者诱导化疗方案：58例（86.6％）采用三药方案，其中47例（70.1％）采用含蛋白酶体抑制剂+免疫调节剂方案（VRD方案45例，67.2％），9例（14.5％）患者采用含1种新药方案。9例（13.4％）采用四药方案（≥2种新药），以D-VRD方案应用最多（3例，4.5％）。

二、自体造血干细胞采集结果

ETO组各项采集指标均优于CTX组（表2）。

**表2:** 依托泊苷（ETO）组与环磷酰胺（CTX）组多发性骨髓瘤患者自体造血干细胞动员结果比较

指标	ETO组（67例）	CTX组（67例）	统计量	*P*值
转换方案［例（%）］	2（3.0）	11（16.4）	*χ*^2^＝6.900	0.009
1天采集成功［例（%）］	59（88.1）	12（17.9）	*χ*^2^＝66.176	<0.001
1天采集优良［例（%）］	30（44.8）	3（4.5）	*χ*^2^＝29.309	<0.001
2天采集成功［例（%）］	66（98.5）	35（52.2）	*χ*^2^＝38.636	<0.001
2天采集优良［例（%）］	55（82.1）	9（13.4）	*χ*^2^＝63.291	<0.001
采集成功［例（%）］	67（100.0）	51（76.1）	*χ*^2^＝18.169	<0.001
采集优良［例（%）］	55（82.1）	14（20.9）	*χ*^2^＝50.244	<0.001
采集天数［d，*M*（范围）］	2（1~3）	2（1~5）	*z*＝−6.651	<0.001
CD34^+^细胞总采集量［×10^6^/kg，*M*（范围）］	7.62（2.26~37.20）	2.73（0.53~9.85）	*z*＝−8.045	<0.001

**注** 转换方案：更换为不同方案化疗动员或加用普乐沙福进行动员

ETO组有2例（3.0％，2/67）患者因采集第1天CD34^+^细胞数<0.8×10^6^/kg而转换方案，均追加普乐沙福行第2天采集，1例获得采集优良，另1例患者采集成功（动员前接受过三线治疗、17个疗程诱导化疗）。CTX组有11例（16.4％）患者因采集1～2 d不理想而转换方案，5例追加普乐沙福再行1～2 d采集，结果3例采集成功（其中1例为二线治疗、6个疗程诱导化疗），2例失败；5例转换为ETO化疗动员，均采集成功，其中2例采集优良（1例为二线治疗、6个疗程治疗后动员）；1例转换为E-CHOP方案化疗动员，采集失败。剔除转换方案患者后两组主要基线特征差异无统计学意义，ETO组（65例）各项采集指标仍优于CTX组（56例），详见表3。

**表3:** 剔除转换方案患者后依托泊苷（ETO）组和环磷酰胺（CTX）组主要基线特征及动员结果比较

指标	ETO组（65例）	CTX组（56例）	统计量	*P*值
年龄［岁，*M*（范围）］	56（39~66）	56（29~64）	*z*＝−0.578	0.563
DS分期［例（Ⅰ期/Ⅱ期/Ⅲ期）］	4/12/49	1/7/48	*χ*^2^＝2.470	0.291
R-ISS分期［例（Ⅰ期/Ⅱ期/Ⅲ期）］	12/39/14	10/36/10	*χ*^2^＝0.301	0.860
含来那度胺化疗［例（%）］	57（87.7）	43（76.8）	*χ*^2^＝2.495	0.114
含达雷妥尤单抗化疗［例（%）］	5（7.7）	3（5.3）	*χ*^2^＝0.266	0.606
动员前疗程［个，*M*（范围）］	4（3~8）	4（2~21）	*z*＝−1.414	0.157
动员前>6个疗程治疗［例（%）］	2（3.1）	3（5.3）	*χ*^2^＝0.395	0.530
动员前缓解程度［例（CR/VGPR/PR/<PR）］	16/30/15/4	17/18/17/4	*χ*^2^＝2.500	0.475
1天采集成功［例（%）］	59（90.8）	12（21.4）	*χ*^2^＝59.653	<0.001
1天采集优良［例（%）］	30（46.2）	3（5.3）	*χ*^2^＝25.243	<0.001
2天采集成功［例（%）］	64（98.5）	33（58.9）	*χ*^2^＝29.568	<0.001
2天采集优良［例（%）］	54（83.1）	10（17.9）	*χ*^2^＝51.356	<0.001
采集成功［例（%）］	65（100.0）	43（76.8）	*χ*^2^＝16.906	<0.001
采集优良［例（%）］	54（83.1）	12（21.4）	*χ*^2^＝46.113	<0.001
采集天数［d，*M*（范围）］	2（1~3）	2（1~5）	*z*＝−5.909	<0.001
CD34^+^细胞总采集量［×10^6^/kg，*M*（范围）］	7.69（2.26~37.20）	2.73（0.53~9.85）	*z*＝−7.691	<0.001

**注** CR：完全缓解；PR：部分缓解；VGPR：非常好的部分缓解

三、造血干细胞动员相关不良反应

134例患者非血液学毒性主要为粒缺伴发热，血液学毒性主要为粒细胞减少、血小板减少，部分患者需要血小板输注。

ETO组有15例（22.4％，15/67）患者发生粒缺伴发热，其中1例肺炎为转换方案患者，未发生血流感染、黏膜炎等严重感染。有8例（11.9％，8/67）患者输注血小板，其中1例为转换方案患者。剔除转换方案的2例患者后，粒缺伴发热的发生率为21.5％（14/65），血小板输注患者占比为10.7％（7/65）。

CTX组有9例（13.4％，9/67）患者发生粒缺伴发热，其中3例为转换方案患者，未发生肺炎、血流感染、黏膜炎等严重感染。有3例（4.5％）患者输注血小板（2例为转换方案的患者）。剔除转换方案的11例患者后，粒缺伴发热的发生率为10.7％（6/56），血小板输注患者占比为1.8％（1/56）。

剔除转换方案的患者，ETO组输注血小板的患者占比高于CTX组［10.7％（7/65）对1.8％（1/56），*χ*^2^＝3.932，*P*＝0.047］，粒缺伴发热发生率差异无统计学意义［21.5％（14/65）对10.7％（6/56），*χ*^2^＝2.555，*P*＝0.110］。

四、自体造血干细胞移植情况

截至2023年8月31日，全部134例患者中117例接受自体造血干细胞移植，其中ETO组63例，CTX组54例。两组患者CD34^+^细胞中位输注量分别为4.62（2.14～19.89）×10^6^/kg、2.62（1.12～5.31）×10^6^/kg（*z*＝−7.206，*P*<0.001），中性粒细胞植入时间分别为11（9～14）d、11（10～14）d（*z*＝−1.972，*P*＝0.049），血小板植入时间分别为11（0～19）d、12（0～34）d（*z*＝−2.095，*P*＝0.035）。有3例患者血小板计数未降到<20×10^9^/L（ETO组2例、CTX组1例）。CTX组有1例患者发生血小板延迟植入（血小板植入时间>21 d）。

五、采集失败的危险因素分析

除动员方案之外，年龄、DS分期、过多的诱导疗程数等因素可能是导致动员、采集失败的原因。采集失败与采集成功患者的主要基线临床特征的比较见表4。

**表4:** 自体造血干细胞采集成功与采集失败多发性骨髓瘤患者的临床特征比较

指标	采集成功（196例）	采集失败（29例）	统计量	*P*值
年龄［岁，*M*（范围）］	55（29~66）	60（41~68）	*z*＝−3.148	0.002
DS分期Ⅲ期［例（%）］	153（78.1）	27（93.1）	*χ*^2^＝3.573	0.059
动员前疗程［个，*M*（范围）］	4（2~21）	4（2~8）	*z*＝−2.055	0.040
动员前化疗疗程>6个［例（%）］	13（6.6）	3（10.3）	*χ*^2^＝0.527	0.468
动员前缓解<PR［例（%）］	9（4.6）	3（10.3）	*χ*^2^＝1.656	0.198
化疗动员方案（例，ETO/CTX）	108/68	0/25	*χ*^2^＝33.156	<0.001
转换方案［例（%）］	20（10.2）	4（13.8）	*χ*^2^＝0.341	0.559

**注** 转换方案：更换为其他化疗动员方案或加用普乐沙福进行动员；PR：部分缓解

对影响干细胞成功的因素进行分析，纳入年龄、性别、疾病分期、诱导治疗方案、诱导治疗疗程数、动员时缓解状态、动员方案以及疾病诊断到动员时间，结果发现年龄增大是导致采集失败的独立不良因素（*OR*＝1.105，95％ *CI* 1.017～1.201，*P*＝0.018）。进一步分析发现，以上因素中，年龄增大是导致未能获得采集优良的不良因素（*OR*＝1.082，95％ *CI* 1.029～1.139，*P*＝0.002）；而相比于ETO动员，采用CTX动员也是获得采集优良的独立不良因素（*OR*＝18.522，95％*CI* 8.976～38.222，*P*<0.001）。

## 讨论

近十几年来，随着蛋白酶体抑制剂、新的免疫调节剂、CD38靶向药物等新药应用于临床，MM的疗效有了很大改观[3]。但不论是在传统药物治疗时代，还是新药时代，自体造血干细胞移植在MM的整体治疗中一直具有独特的作用，其地位仍然不可取代[4]。目前国内外指南均推荐所有适合移植的患者进行自体造血干细胞移植[6]–[7]。由于单用化疗动员或者G-CSF动员，自体造血干细胞动员的效果均不理想[8]，一般采用联合方案（即化疗+G-CSF或普乐沙福+G-CSF）进行动员[9]–[10]。

因价格因素影响，我中心大部分患者初始动员选择了化疗+G-CSF的方案。常用的动员化疗方案有CTX化疗、ETO化疗等。文献报道ETO剂量为（375～2 400）mg/m^2^ [11]，我国学者采用的剂量大多为（1 200～1 600）mg/m^2^。我中心经预研究，选择了375 mg/m^2^×2 d这种用法。从本组病例的结果来看，ETO化疗动员、采集2天的成功率和优良率均优于CTX化疗动员，需要的采集天数少于CTX组，转换方案的患者占比也较低，提示ETO化疗动员的效果更好。采集方面的优势，使ETO组患者移植时有更高的回输干细胞数，粒细胞及血小板植入更快，减少发生严重并发症引起血小板延迟植入的风险。安全性方面，剔除转换方案的患者，两组发生粒缺伴发热的比例差异无统计学意义，但ETO组需要血小板输注的患者占比高于CTX组。

本研究与李菁等[12]曾报道的相似，均证实ETO化疗动员比CTX化疗动员更为高效。但有以下不同：①本研究ETO剂量较小，在动员效率相当的情况下，感染及输血等不良反应减少。②纳入本研究的患者不局限于新诊断MM，提示ETO化疗动员仍有优势。③基于真实世界的状况，本研究观察了动员过程中的方案转换问题，ETO组需要挽救转换的比例低，而且追加普乐沙福即能获得良好的结果，在有普乐沙福追加的情况下，采集成功率几乎能达100％，优良率达到85.8％；相较而言，初始选择CTX化疗动员的患者（CTX组），需要挽救治疗的比例高，而且追加普乐沙福的效果并不突出，改为ETO化疗或者含普乐沙福的方案进行二次动员才有良好的结果。④ETO化疗用于挽救动员（有过CTX动员失败史）也有良好效果（7例均成功，其中4例达优良）。

目前比较公认的动员、采集失败的高危因素有年龄大、骨髓广泛受累、强烈治疗及多疗程治疗史，等[8],[13]–[14]。本研究中，通过多因素分析发现，ETO化疗动员是获得优良采集结果的正性因素（不受年龄、DS分期、诱导治疗疗程数等影响），而年龄大是负性因素。从采集失败患者的特征来看，疗程数也是有统计学意义的差异性特征。

本研究提示，小剂量ETO化疗动员效果优于CTX，接近实现一次动员即成功的目标，少数第1天采集不理想的患者，追加普乐沙福可取得良好效果。本研究采用的ETO剂量仍有一定的感染发生率及血小板输注率，后续需要进一步研究优化动员方案，实现更高效、安全、经济的动员。
